# Enhancing Adolescent HIV Screening: A Quality Improvement Approach Using Point-of-care Testing in an Outpatient Pediatric Setting

**DOI:** 10.1097/pq9.0000000000000942

**Published:** 2026-10-28

**Authors:** Graydon Dietrich, Natasha Thomas, Alexandria Grellmann, Gabrielle Pina, Rachel Davidge, Jerard Tambunan, Mona Abutouk

**Affiliations:** From the * Loma Linda University School of Medicine, Loma Linda, Calif.; † Department of Pediatrics, Loma Linda University, Loma Linda, Calif.

## Abstract

**Introduction:**

Routine HIV testing remains poorly implemented nationwide, despite national recommendations. Half of adolescents who are HIV positive do not know their status. This quality improvement project aimed to increase the rate of routine HIV screening during well-child checks (WCCs) for eligible adolescents 15–18 years of age with no prior lifetime HIV test to greater than 50% within 1 year through implementation of point-of-care (POC) oral swab HIV testing at a pediatric outpatient clinic affiliated with Loma Linda University in Moreno Valley, Calif.

**Methods:**

All adolescent patients 15–18 years of age presenting for WCC who had not yet been tested once in their lifetime for HIV were eligible. We conducted 6 plan-do-study-act cycles from August 2023 to September 2025, including initial and subsequent implementation cycles. Key drivers included standardizing the process to facilitate HIV testing in the clinic, improving provider awareness, providing standardized information on patient eligibility, and providing patient and family education on HIV screening. The primary intervention was implementing oral swab POC testing during WCC visits. Other interventions included creating an educational curriculum for physicians on HIV screening guidelines, developing multilingual resources for families on the importance of HIV screening, and using a multidisciplinary team to integrate testing into clinic workflow. The primary outcome measure was the monthly percentage of adolescents 15–18 years of age who were screened for HIV via the POC swab during their WCC.

**Results:**

Before implementation, the average percentage of eligible patients screened from January 2023 to July 2023 was 29%. Screening rates increased from 29% to 51% among eligible adolescents over the first eight months. With implementation of the POC oral swab HIV test, screening rates increased from 51% to 56% over an additional 8 months. During the later implementation cycles (December 2024 to September 2025), monthly screening rates generally exceeded the project goal of 50%, averaging 64%. Eligibility was based on age, visit type, and prior lifetime HIV testing status. No reactive or positive HIV test results were identified during the study period. Potential balancing measures included additional time needed during clinical visits, false-positive results, and the potential workflow burden on clinical staff.

**Conclusions:**

Improving HIV screening among adolescents is feasible through a multicomponent quality improvement strategy that incorporates POC rapid antibody testing. This type of testing is painless, can be performed quickly during clinic visits, and improves patient confidentiality because results are available at the time of the visit rather than by phone or email. Medicaid covers POC testing for HIV. This makes this form of testing feasible in many clinics.

## INTRODUCTION

Pediatricians play a key role in identifying HIV infection by offering routine HIV testing. Although there has been great progress in treatment for people living with HIV/AIDS, many adolescents still believe they are not at risk for HIV and have never been tested, with almost half of adolescents who have HIV unaware of their diagnosis.^1^ In 2023, there were 39,201 new HIV diagnoses of patients 13 years and older in the United States and 6 territories and freely associated states. Among these patients, 18% were 13–24 years of age.^2^

Adolescents have also been shown to have lower rates of engagement in care and lower rates of viral suppression compared to other age groups.^3^ National organizations including the Centers for Disease Control and Prevention (CDC), the American Academy of Pediatrics (AAP), and the United States Preventive Services Task Force recommend routine HIV screening beginning in adolescence. The CDC recommends opt-out screening for all patients 13–64 years of age at least once, regardless of risk factors. The AAP recommends screening for all 16- to 18-year-olds residing in communities with an HIV prevalence equal to or greater than 0.1%. The United States Preventive Services Task Force recommends screening at least once between the ages of 15 and 65, regardless of risk factors.^4–6^

Despite these recommendations, routine HIV screening in adolescent populations remains limited.^7,8^ In our outpatient clinic in Moreno Valley, Calif., approximately 29% of adolescents 15–18 years of age were screened for HIV. Successful implementation of routine HIV screening has mainly been seen in the emergency department setting, which may be attributed to on-site access to laboratory testing and sexually transmitted infection (STI) testing protocols.^9^ A key barrier to screening adolescents for HIV during clinic well-child check (WCC) includes barriers to obtaining HIV testing at a laboratory. Traditionally, this requires a clinician to order the test, patient transportation to the laboratory site, a blood draw, insurance approval, and a waiting period before receiving results. Studies show that steps outside the clinic visit decrease the likelihood of completing screening.^4,7^

Point-of-care (POC) HIV testing provides results within minutes and removes the need for additional visits. Prior studies have shown that rapid testing in clinics improves screening uptake and patient acceptance.^7^ These findings suggest that increasing the utilization of POC HIV testing during clinic visits may improve screening completion. However, there are limited studies evaluating the use of POC HIV oral swab testing in routine adolescent well-child visits in the outpatient setting. Additionally, the literature shows that adolescents prefer rapid POC testing over traditional laboratory-based testing, further supporting POC testing in this population.^10^

This quality improvement initiative aimed to improve HIV screening rates in eligible adolescents with no prior lifetime HIV testing 15–18 years of age presenting for a WCC to greater than 50% within 1 year of implementation through the integration of POC HIV oral swab testing into routine WCCs.

## METHODS

### Context

This study was conducted as a quality improvement initiative in a pediatric outpatient clinic affiliated with Loma Linda University in Moreno Valley, Calif. Moreno Valley is home to a large, diverse population in Southern California’s Inland Empire. The surrounding community of Riverside County has over 2 million residents. It serves approximately 817,448 children, with a diverse demographic makeup that includes a large proportion of Hispanic and Latino individuals.^11^ This population also has a high percentage of households that speak a language other than English. Additionally, during 2023–2024, Riverside County had approximately 938,000–966,000 Medi-Cal enrollees per quarter, out of a county population of approximately 2.4 million, representing roughly 38%–40% of the total county population enrolled in Medi-Cal.^12^ This further supports the feasibility of implementing POC HIV oral swab testing because it is covered under Medicaid. In California, there were 740 newly diagnosed HIV cases among individuals 13–24 years of age in 2023.^13^ Riverside County is a region with a high HIV prevalence rate of 481.8 per 100,000.^14^ This prevalence exceeds the AAP threshold of 0.1% (1 per 1,000), supporting the use of routine, opt-out HIV screening in this population.^5^

The clinic provides routine WCCs to patients aged 0–18 and sees about 43,630 pediatric patients annually, including 20,900 WCCs. The clinic staff includes 10 attending physicians, 12 pediatric residents, and 12 MAs. Adolescent WCCs follow standard preventive guidelines such as growth screening, blood pressure, vision and hearing, depression, and sexually transmitted diseases.

Based on current screening recommendations, adolescents aged 15–18 presenting for a WCC who had never been tested for HIV were selected as the eligible target population to increase screening to greater than 50% within 1 year of implementation. We determined prior lifetime HIV testing status based on the health-system electronic medical record (EMR) and patient or family report. Our clinic used the OraQuick ADVANCE HIV-1/2 rapid antibody test, manufactured by OraSure Technologies, with 92% sensitivity and 99.98% specificity. The implementation of POC HIV oral swab testing was done to address factors such as barriers to laboratory-based screening, limited patient education on routine HIV screening, no standardized HIV screening workflow, and limited awareness of national HIV guidelines.

### Interventions

Before implementation, the team evaluated current clinic workflows and quality measures to identify potential barriers to HIV screening. A qualitative evaluation among physicians and staff, consisting of structured discussions and workflow mapping, showed key gaps such as a lack of standardized workflow, limited provider awareness, inconsistent knowledge of patient eligibility, and limited education for patients and families on HIV screening. In collaboration with clinic staff, we created a workflow to integrate HIV screening into routine visits. The clinic developed educational materials and workflow resources for implementation, which are publicly available on the Loma Linda University PCT Pediatrics program website (https://sites.google.com/llu.edu/pct-pediatrics/clinic/clinic-qi-projects/hiv). Interventions addressed barriers to screening and included developing a physician-education curriculum on HIV screening guidelines, creating multilingual educational materials for patients and families, forming a multidisciplinary team including physicians, nurses, and clinic staff, and implementing POC HIV oral swab testing during well-child visits. We selected these interventions to improve provider awareness, reduce patient-related barriers, and integrate testing into clinic workflow.

### Quality Improvement Framework

We created a fishbone diagram to analyze local factors contributing to low HIV screening rates (Fig. 1). We used the identified factors to develop a key driver diagram with our SMART aim, key drivers, and interventions, along with associated plan-do-study-act (PDSA) cycles (Fig. 2). We used multiple PDSA cycles to implement and refine the intervention. These PDSA cycles helped identify screening barriers, improve workflow integration, reinforce provider adherence, and monitor changes in screening rates over time. Adjustments to clinic workflow included integrating nursing and medical-assistant involvement to identify eligible patients, provide education, obtain verbal patient consent, and facilitate POC testing during the visit. Figure 3 shows the revised clinic workflow and the steps changed from the original workflow.

**Fig. 1.**
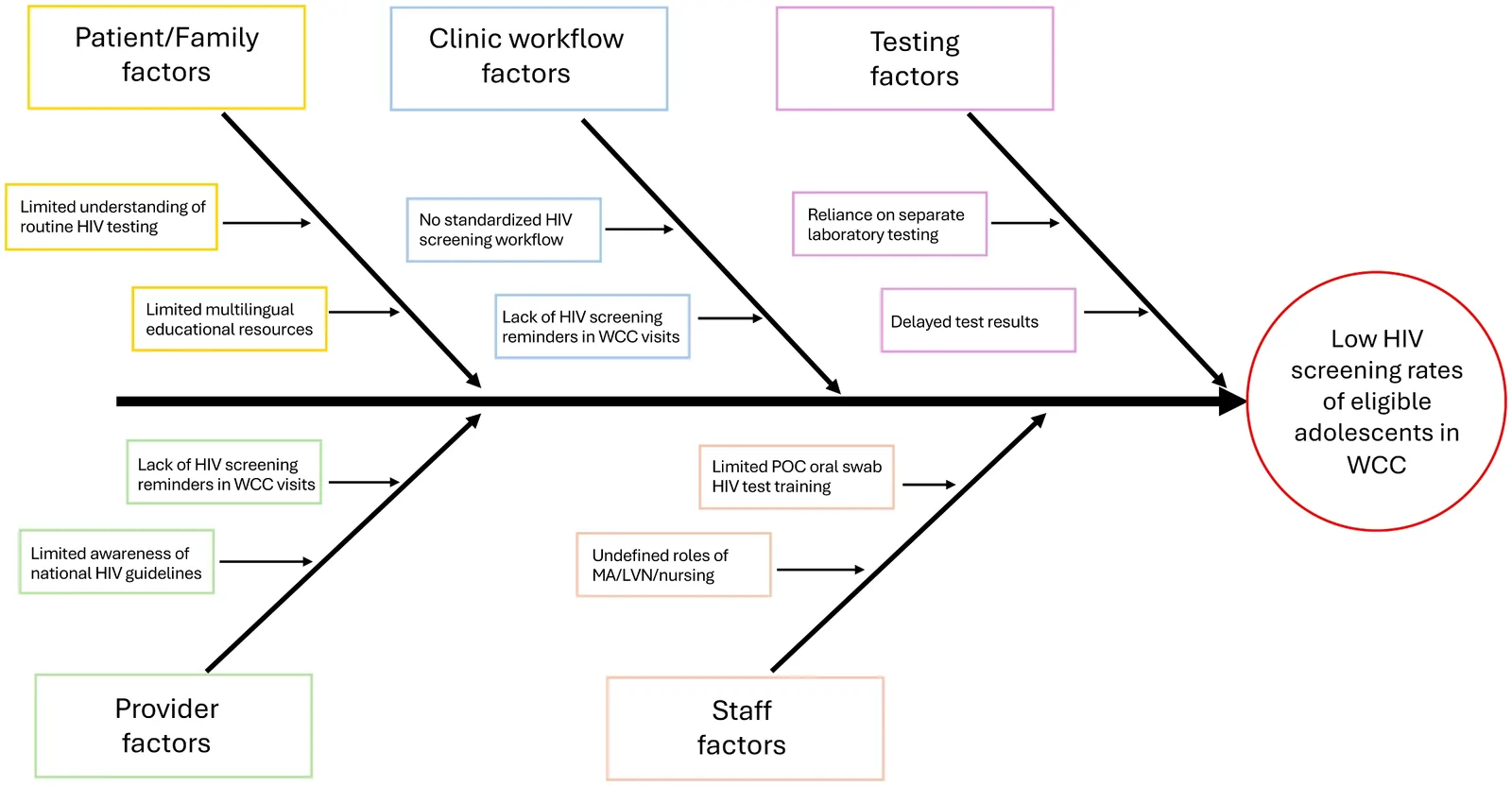
Fishbone diagram identifying factors such as provider, staff, clinic workflow, patient/family, and testing contributing to low HIV screening rates among eligible adolescents 15–18 years of age who have never had an HIV test during a WCC.

**Fig. 2.**
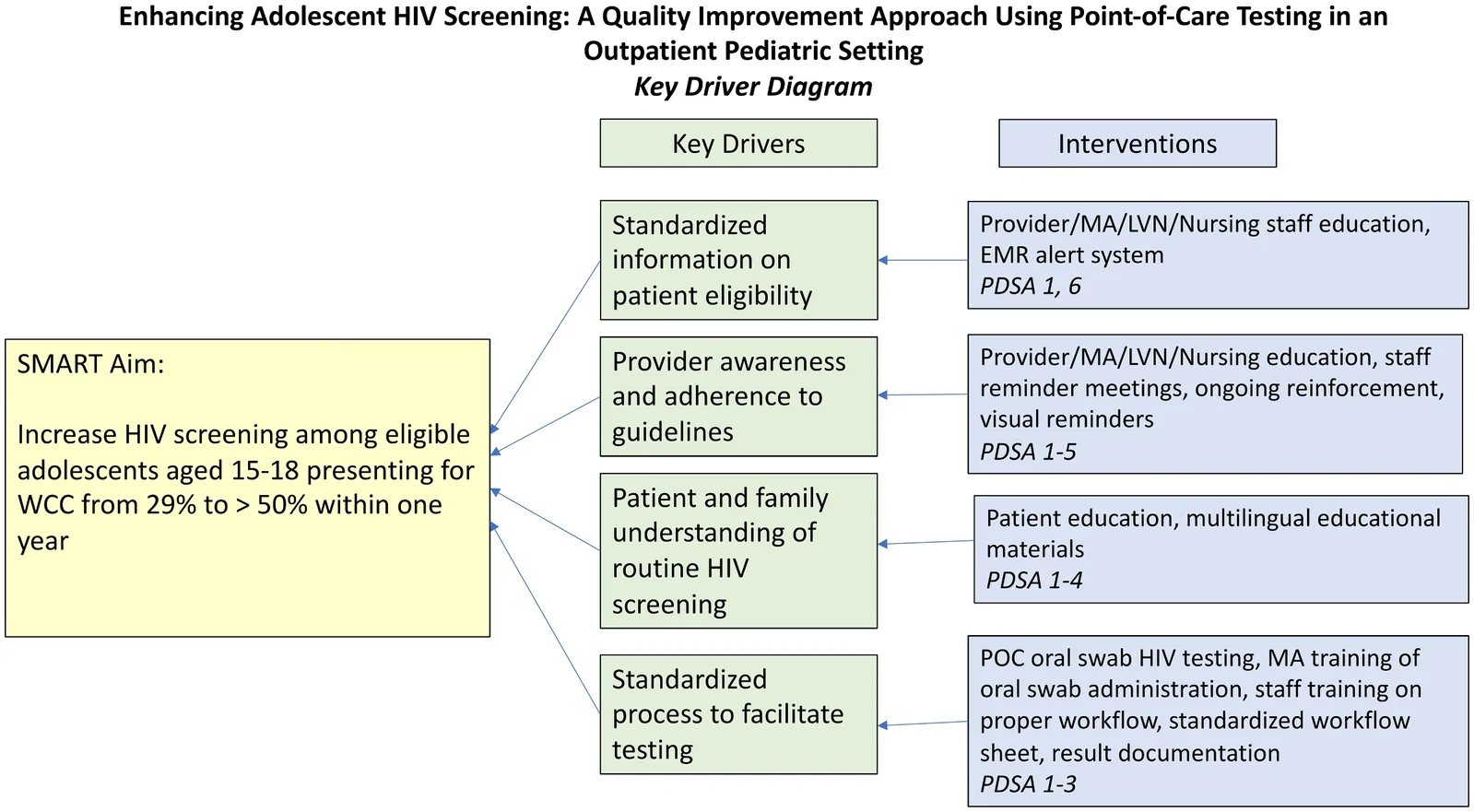
Key driver diagram including the aim of the study as a SMART goal, key drivers, and interventions implemented in PDSA cycles.

**Fig. 3.**
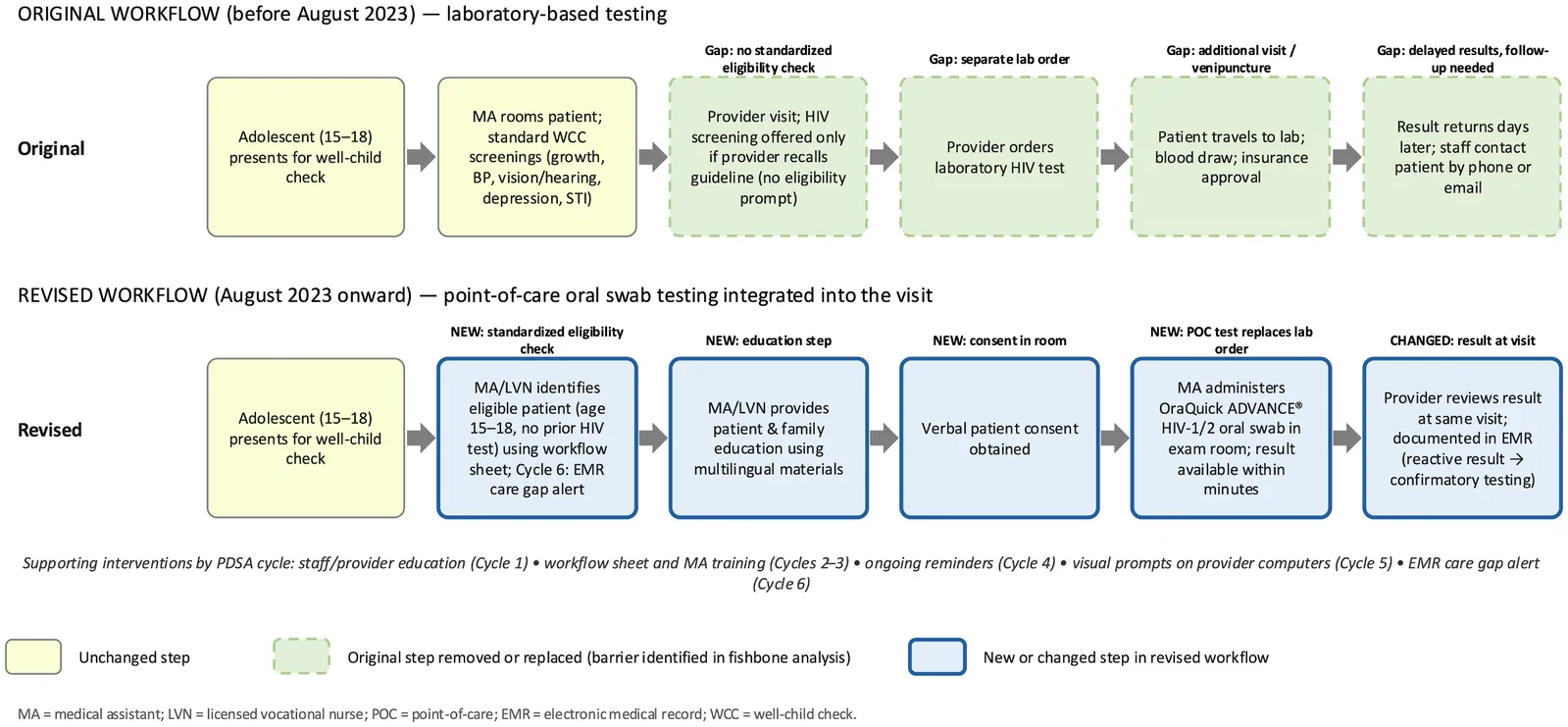
Revised clinic workflow map for HIV screening during adolescent WCC, highlighting changes from the original workflow: identifying eligible patients by MAs and nursing staff, patient and family education, verbal patient consent, POC oral swab HIV testing during the visit, and result documentation.

### Cycle 1 (August 2023–September 2023)

The initial phase focused on introducing the quality improvement initiative to clinic staff. The team held a house-staff meeting with medical assistants (MAs), licensed vocational nurses, and nursing staff to review the project’s purpose, patient population demographics, and the importance of routine HIV screening. During this phase, the team assessed baseline clinic workflows and identified key barriers to screening, including a lack of standardized processes and limited provider awareness. This phase also indicated when POC HIV oral swabs became available for use in the clinic.

### Cycle 2 (October 2023–December 2023)

After initial implementation, a reminder meeting with all clinic MAs reinforced the importance of routine HIV screening. We developed a standardized workflow sheet to guide staff through the screening process and reduce variability during patient visits. Staff training ensured proper use of the workflow and POC HIV testing equipment.

### Cycle 3 (January 2024–June 2024)

The team held additional house-staff meetings to reinforce adherence to screening protocols and address challenges identified during early implementation. Workflow sheets were modified to improve usability and better integrate into clinic flow. The team held targeted training sessions with MAs to demonstrate proper administration of the oral rapid HIV swab and increase staff confidence in POC testing.

### Cycle 4 (July 2024–January 2025)

The team reinforced the intervention through additional staff meetings and reminders. These efforts aimed to maintain adherence to screening protocols and sustain improvements in screening rates.

### Cycle 5 (February 2025–May 2025)

To further improve adherence, the team introduced visual reminders by placing prompts on provider workroom computers. These reminders served as real-time cues during patient encounters, reducing missed opportunities due to provider oversight.

### Cycle 6 (June 2025–September 2025)

In the final phase, we implemented an EMR-based intervention within the EPIC system (Verona, Wis.). A care gap alert was created to notify providers when eligible patients had not yet been screened for HIV. This automated reminder improved the identification of eligible patients, reduced reliance on memory or manual workflow steps, and supported the long-term integration of screening into clinic practice.

### Measures

The study aimed to increase HIV screening rates among eligible adolescents aged 15–18 to greater than 50% within 1 year of implementation. A baseline average HIV screening rate in eligible adolescents from January 2023 to July 2023, collected retrospectively from the EMR, was 29%; the primary outcome measure was the monthly percentage of adolescents aged 15–18 screened for HIV during their WCC visit. We tracked screening rates from January 2023 to September 2025, including the preimplementation period, the main implementation period, and later implementation cycles. Possible balancing measures not formally assessed during the study include additional time needed during clinical visits, false-positive results, and potential workflow burden on clinical staff. We informally reviewed time to patient rooming during early implementation and found minimal change; we did not collect formal balancing-measure data.

### Data Analysis

We calculated screening rates as the proportion of eligible adolescents who received HIV screening during WCC visits. We tracked data monthly to assess trends in screening rates over time and evaluate the intervention’s impact. Baseline data from January 2023 to July 2023 were obtained retrospectively through EMRs. We summarized rates for the preimplementation period, early postimplementation period, late postimplementation period, and a later implementation period (December 2024 to September 2025) (Table 1). We displayed monthly screening percentages using a run chart with a goal of greater than 50% and the baseline median from the preimplementation period (Fig. 4). The run chart was used descriptively to assess trends and sustainability of improvement over time in accordance with SQUIRE 2.0. The run chart was interpreted using standard run chart rules: the median of the baseline period (January to July 2023) was extended as the reference line, a shift was defined as 6 or more consecutive points on one side of the median, a trend as 5 or more consecutively increasing or decreasing points, and astronomical points as values clearly unlike the rest of the series.

**Table 1.** HIV Screening Rates Before and After Implementation of POC Testing

Study Period	Eligible Adolescents	Screened (n/N)	Screening Rate (%)
Preimplementation (January 2023–July 2023)	276	80/276	29
Early postimplementation (August 2023–March 2024)	320	164/320	51
Late postimplementation (April 2024–November 2024)	351	196/351	56
Later implementation (December 2024–September 2025)	524	335/524	64

**Fig. 4.**
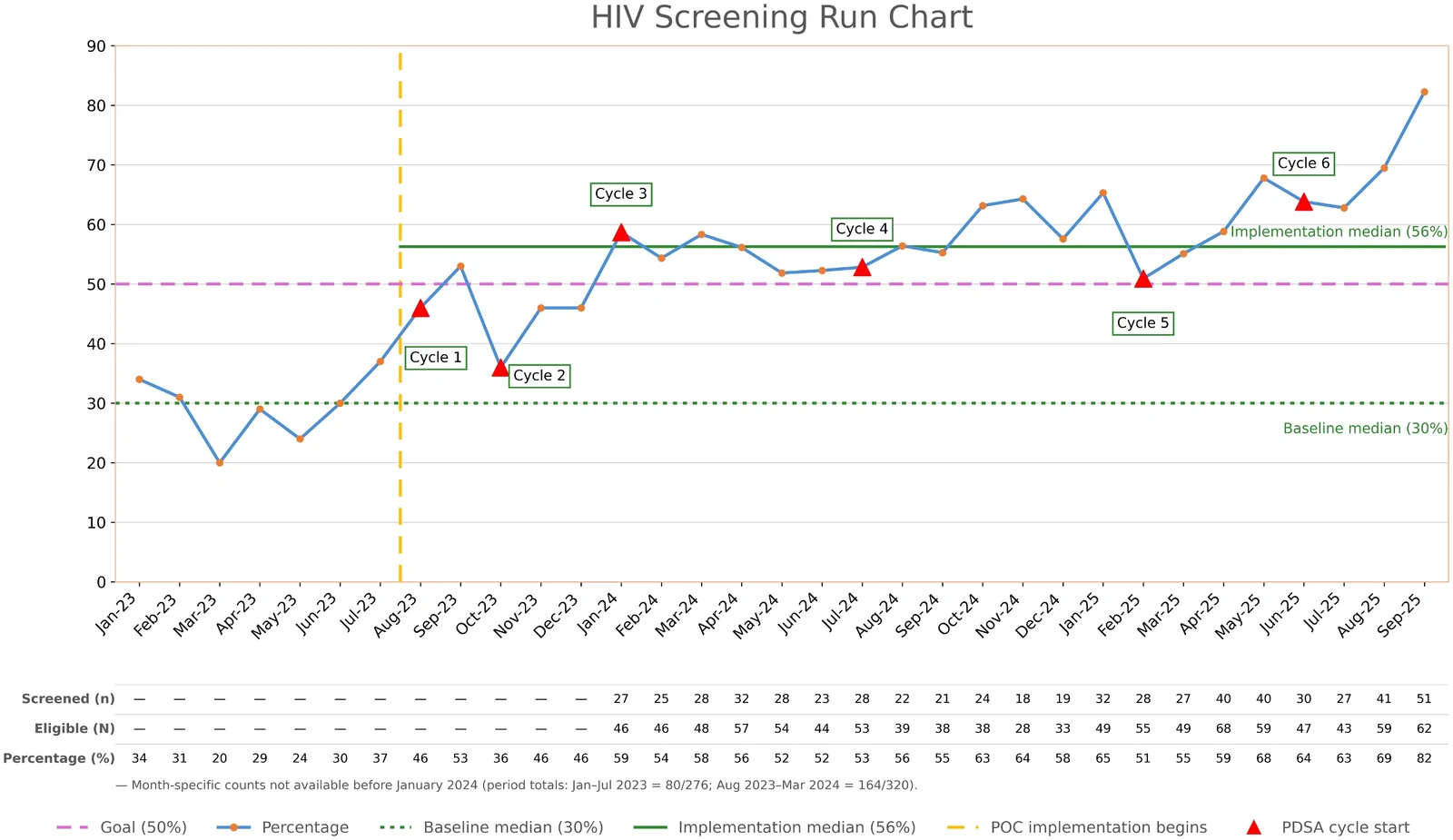
Run chart of the monthly percentage of eligible adolescents 15–18 years of age screened for HIV during WCC. PDSA cycle interventions are labeled as cycles 1–6. Cycle 1 (August 2023): Staff education, baseline workflow assessment. Cycle 2 (October 2023): Standardized workflow sheet, staff training. Cycle 3 (January 2024): Reinforcement meetings, MA training on oral swab. Cycle 4 (July 2024): Ongoing staff reminders, protocol adherence. Cycle 5 (February 2025): Visual computer prompts for providers. Cycle 6 (June 2025): EMR-based EPIC care gap alert. Key: Orange dots with a connecting blue solid line represent plotted points of the monthly routine HIV screening rate. The purple dashed line represents the goal rate of 50%. The green dotted line represents the baseline median of 30% for January 2023–July 2023, before the implementation of POC HIV testing. The green solid line represents the implementation period median of 56% from August 2023 to September 2025. The vertical dashed line marks the start of implementation in August 2023. Red triangles mark the start of each PDSA cycle. The table below the *x*-axis shows monthly screened (n), eligible (N), and percentage; month-specific counts were not available before January 2024.

### Ethical Considerations

This project was reviewed by the clinical attending physicians and department leadership and determined to be quality improvement rather than human-subjects research; a formal institutional review board determination was not obtained, in accordance with institutional policy governing quality improvement activities.

## RESULTS

Before implementation, the year-to-date percentage of eligible patients screened from January 2023 to July 2023 was 29% (80/276). Within 8 months of implementation, from August 2023 to March 2024, the percentage screened increased to 51% (164/320), a 22-percentage-point increase. In the subsequent 8 months, from April 2024 to November 2024, the rate increased to 56% (196/351), a 27-percentage-point increase from baseline. On the run chart (Fig. 4), the baseline median was 30%. Beginning in August 2023, all 26 subsequent monthly rates were above the baseline median, meeting the shift rule (6 or more consecutive points on one side of the median) and indicating nonrandom, special-cause variation coinciding with implementation; the median during the implementation period was 56%. One trend of five consecutively increasing points was identified from May 2023 to September 2023, spanning the start of implementation. No other trends or astronomical points were identified, and month-to-month fluctuations within each period were consistent with random variation. Rates were generally above the project goal of greater than 50%. During the later implementation cycles from December 2024 to September 2025, average monthly rates largely remained above the goal, with a period average of 64%. These findings show increased screening rates after implementing the bundled interventions, including POC HIV testing, and suggest that integrating testing into clinic workflow was associated with improved screening completion. Because we implemented multiple interventions, we could not isolate the contribution of any single intervention, including POC testing. Time to patient rooming, informally reviewed during early implementation as an indicator of added nursing workload, showed minimal change; we did not formally track balancing measures. No reactive or positive HIV test results were identified among screened adolescents during the study period; consequently, no false-positive results occurred.

Screening rates across study periods are summarized in Table 1.

## DISCUSSION

Improving HIV screening among adolescents is feasible through a multicomponent quality improvement strategy that incorporates POC rapid antibody testing. Our study showed improved screening rates after implementing POC HIV oral swab testing, along with staff education, workflow standardization, and provider reminders during WCC.

A key barrier to screening adolescents for HIV is reliance on laboratory-based testing. Before implementation, testing required a separate laboratory visit, adding steps and delaying results. This process likely contributed to missed screening opportunities. The introduction of POC testing removed this barrier by allowing testing during the clinic visit, with results available at the time of the encounter. This change simplified the screening process and likely contributed to improved completion rates, although we cannot separate its independent effect from the other interventions.

Our findings are consistent with prior studies demonstrating that the implementation of rapid HIV and STI testing can significantly increase screening rates in pediatric populations. Previous outpatient pediatric studies have shown screening increasing from approximately 5% to 41% and from 30% to 80% after implementing a similar quality improvement intervention involving POC HIV testing, PDSA cycles, and staff education.^7,8^ One study was able to diagnose patients with HIV and connect them with care the same day.^8^ In a pediatric ED, implementation of POC HIV testing increased HIV testing offers from 3.6% to more than 87%.^9^ Additionally, EMR-based alerts have been found to improve STI screening rates in young adults in the primary care setting.^15^ Together, these findings show that rapid POC HIV testing, along with staff and patient education, standardized clinic workflow, and EMR-based alerts, can be used in various pediatric settings to increase HIV screening.

This study builds on this work by showing that screening rates can be further improved and remain above 50% in an outpatient pediatric setting. Screening rates increased from a baseline of 29%–51% during early implementation, remained at 56% during late postimplementation, and exceeded the goal at 64% during the later implementation period. The additional PDSA cycles 5 and 6, which took place from February 2025 to September 2025, represent our ongoing efforts toward long-term sustainability. Because we introduced new interventions during this period, it reflects an ongoing implementation phase rather than a true sustainability period, and the improvement from 56% to 64% may reflect the additional interventions rather than sustained performance alone. Additionally, this initiative was conducted in a largely Hispanic and Latino, Medicaid-insured, and language-diverse population in a high-HIV-burden county, which helps expand the generalizability of POC HIV testing interventions to underserved communities.^11,12,14^ Overall, these findings support the idea that outpatient pediatric HIV screening can be improved not only through test availability but also through deliberate workflow redesign, repeated reinforcement, and system-level supports.

POC HIV testing has several advantages. This type of testing is painless, can be performed quickly during clinic visits, and improves patient confidentiality because results are available at the time of the visit rather than by phone or email. This approach also reduces reliance on patient follow-through, which can reduce missed screening opportunities. Medicaid covers POC testing for HIV, which allows this intervention to be implemented in many clinical settings. Routine HIV screening in adolescents remains an important public-health priority, and integration of POC testing into well-child visits may improve adherence to national screening recommendations.^4–6^ No reactive or positive HIV test results were identified during the study period. Future efforts should focus on establishing a treatment pathway for adolescents who screen positive for HIV. Also, improving access to HIV prevention services such as pre-exposure prophylaxis for patients who are at high risk would be beneficial.

### Limitations

This study was conducted at a single center, which may limit generalizability. This was a quality improvement study and was not randomized. Provider adherence to screening recommendations may have varied during the study period. No positive HIV test results were identified during the study period; therefore, linkage to care following a positive screen could not be evaluated. The study did not formally assess potential balancing measures such as time required during clinic visits or workflow burden on clinical staff; an informal review of time to rooming during early implementation showed minimal change, but these data were not systematically collected and could not be retrospectively estimated. Because this was a quality improvement strategy with multiple interventions, we cannot determine which intervention contributed most to the observed increase in screening rates. Additionally, documentation limitations made it difficult to determine whether HIV screening was offered but declined versus not offered, which may have impacted measured screening rates; refusal data were not consistently documented in the EMR during the study period, so refusal rates could not be reported. Finally, because the EMR-based care gap alert was introduced during the final months of the study period, the study did not capture a true sustainability period after all interventions were completed.

## CONCLUSIONS

Improving HIV screening among adolescents is feasible through a structured quality improvement strategy that integrates POC testing with staff education, workflow standardization, visual prompts, and EMR-based decision support. This approach reduces barriers to testing, improves patient confidentiality, and makes results available at the time of the visit. Future efforts should expand screening to additional visit types and improve linkage to prevention services for all adolescent patients. Efforts should also document screening offers, refusals, and completed tests to better evaluate the causes of incomplete screening. Balancing measures, such as visit duration and staff workload, should be prospectively defined and tracked. This initiative may serve as a model for outpatient pediatric clinics serving diverse and linguistically varied adolescent populations seeking to improve adherence to national HIV screening guidelines.

### Compliance with Ethical Standards

Clinical attending physicians and department leadership reviewed this project and determined it to be quality improvement rather than human-subjects research; therefore, the authors did not obtain a formal institutional review board determination, in accordance with institutional policy governing quality improvement activities.

## ACKNOWLEDGMENTS

The authors thank the nursing staff, MAs, and clinical team at the Loma Linda University Moreno Valley pediatric outpatient clinic for their engagement in this quality improvement initiative. Educational materials and workflow resources developed for this initiative are available through the Loma Linda University PCT Pediatrics program (https://sites.google.com/llu.edu/pct-pediatrics/clinic/clinic-qi-projects/hiv).

Artificial intelligence–assisted tools, specifically Claude (Anthropic), supported literature identification, editing for clarity and grammar, and reference formatting during manuscript preparation; the authors independently verified all sources. All clinical data, statistical analyses, and conclusions represent the original work of the authors.
